# Anatomy of BioJS, an open source community for the life sciences

**DOI:** 10.7554/eLife.07009

**Published:** 2015-07-08

**Authors:** Guy Yachdav, Tatyana Goldberg, Sebastian Wilzbach, David Dao, Iris Shih, Saket Choudhary, Steve Crouch, Max Franz, Alexander García, Leyla J García, Björn A Grüning, Devasena Inupakutika, Ian Sillitoe, Anil S Thanki, Bruno Vieira, José M Villaveces, Maria V Schneider, Suzanna Lewis, Steve Pettifer, Burkhard Rost, Manuel Corpas

**Affiliations:** Bioinformatik, Technische Universität München, Garching, Germany; TUM Graduate School, Center of Doctoral Studies in Informatics and its Applications, Technische Universität München, Garching, Germany and Biosof LLC, New York, United States; Bioinformatik, Technische Universität München, Garching, Germany and TUM Graduate School, Center of Doctoral Studies in Informatics and its Applications, Technische Universität München, Garching, Germany; Bioinformatik, Technische Universität München, Garching, Germany; Bioinformatik, Technische Universität München, Garching, Germany; Bioinformatik, Technische Universität München, Garching, Germany; Molecular and Computational Biology, University of Southern California, Los Angeles, United States; Web and Internet Science, University of Southampton, Southampton, United Kingdom; Banting and Best Department of Medical Research, Donnelly Centre for Cellular and Biomolecular Research, University of Toronto, Toronto, Canada; Linkingdata I/O LLC, Austin, United States; European Molecular Biology Laboratory-European Bioinformatics Institute, Cambridge, United Kingdom; Bioinformatics Group, Department of Computer Science and Centre for Biological Systems Analysis, University of Freiburg, Freiburg, Germany; Web and Internet Science, University of Southampton, Southampton, United Kingdom; Institute of Structure and Molecular Biology, University College London, London, United Kingdom; The Genome Analysis Centre, Norwich, United Kingdom; School of Biological and Chemical Sciences, Queen Mary University of London, London, United Kingdom; Max Planck Institute of Biochemistry, Planegg, Germany; The Genome Analysis Centre, Norwich, United Kingdom; Lawrence Berkeley National Laboratory, Berkeley, United States; School of Computer Science, The University of Manchester, Manchester, United Kingdom; Bioinformatik, Technische Universität München, Garching, Germany; TUM Graduate School, Center of Doctoral Studies in Informatics and its Applications, Technische Universität München, Garching, Germany and Biosof LLC, New York, United States; The Genome Analysis Centre, Norwich, United Kingdom

**Keywords:** cutting edge, open source, JavaScript, community, bioinformatics, visualization, none

## Abstract

BioJS is an open source software project that develops visualization tools for different types of biological data. Here we report on the factors that influenced the growth of the BioJS user and developer community, and outline our strategy for building on this growth. The lessons we have learned on BioJS may also be relevant to other open source software projects.

**DOI:** http://dx.doi.org/10.7554/eLife.07009.001

## Introduction

**Figure 1.**
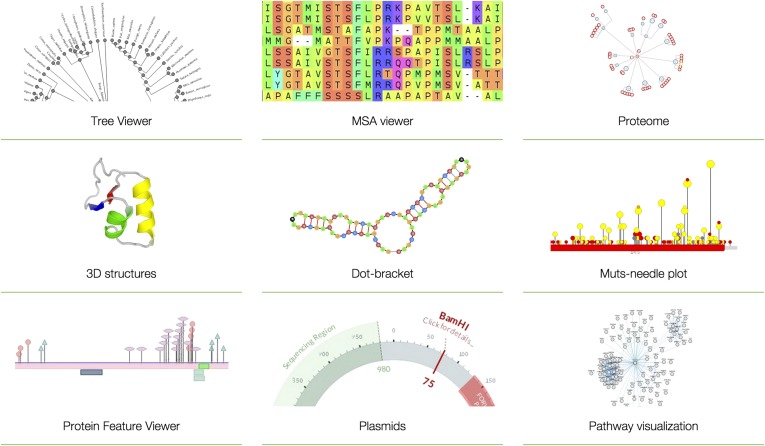
Examples of BioJS tools. Tree Viewer (visualization of phylogeny data in a tree-like graph); MSA Viewer (visualization and analysis of multiple sequence alignments); Proteome (multilevel visualization of proteomes in UniProt; The UniProt Consortium, 2015); 3D structures (visualization of protein structures); Dot-bracket (visualization of RNA secondary structures); Muts-needle plot (presentation of mutation distribution across protein sequences). Protein Feature Viewer (visualization of position-based annotations in protein sequences); Plasmids (visualization of DNA plasmids); Pathway visualization (visualization of data from Pathway Commons; Cerami et al., 2011). Note that all visualization tools are native to the browser and do not require any specialized software (such as Adobe flash, Java Virtual Machine or Microsoft Silverlight) to be installed or loaded. **DOI:** http://dx.doi.org/10.7554/eLife.07009.002

BioJavaScript (BioJS; http://biojs.net/) was set up to meet a need for an open source library of reusable components to visualize and analyse biological data on the web (Figure 1; Corpas et al., 2014). These components are discrete modules that can be reused, extended and combined to meet a particular visualization need. Unlike proprietary (or closed-source) systems, which are typically distributed as ‘executable’ files under restrictive licenses, open source software projects make their source code freely available under a permissive license (Millington, 2012; Balch, et al., 2015). This allows other users to modify, extend and re-distribute the software with few restrictions and at no cost to other users.

However, the fact that the community is allowed and encouraged to contribute to an open source software project is no guarantee that they will contribute. It is certainly not a sufficient condition for ensuring sustainability: repositories of open source software such as GitHub (https://github.com) and SourceForge (http://sourceforge.net) are littered with abandoned projects that have failed to gain the support needed to survive beyond the originator's initial enthusiasm and/or funding.

In the case of BioJS we were acutely aware of the need to gain buy-in from the community for two reasons:

1. To fulfil the vision of a suite of tools capable of displaying diverse biological data requires expertise and capacity that is well beyond that of any individual group of developers working in isolation.
2. To encourage users to spend time integrating BioJS tools into their websites and applications, the project has to have, and be seen to have, a potential lifespan far beyond that of its initial funding.

Predicting the success of projects is hard, and there is nothing in the nature of open source initiatives that makes this any easier. There have been attempts to quantify success in open source software (see, e.g., the metrics proposed by Crowston et al., 2003), but these have gained little traction. More useful, we feel, have been articles that provide pragmatic advice to would-be open software developers, such as ‘Ten simple rules for the open development of scientific software’ (Prlic and Procter, 2012). Rather than repeating or re-working the general-purpose recommendations of others, here we describe what we have learned from the BioJS project.

## Project evolution

BioJS was initially developed in 2012 through a collaboration between the European Bioinformatics Institute (EMBL-EBI) and The Genome Analysis Centre (TGAC). The project began as a set of individual graphical components deposited in a bespoke online registry. Since then it has expanded into a community of 41 code contributors spread across four continents, a Google Group forum with more than 150 members (https://groups.google.com/forum/#!forum/biojs), and 15 published papers (as of May 2015). The project's first paper, published in 2013 (Gomez, et al., 2013), has been cited 31 times to date.

The BioJS registry (http://biojs.io) offers a modern platform for fast and customizable access to components. The registry provides a centralized resource where deposited components can be discovered, tested and downloaded at the push of a button. BioJS components are organized as packages. This modular approach reduces duplication of effort and has been used by other projects, such as BioGem (Bonnal, et al., 2012). Several recognized projects and institutions have already shown commitment to BioJS by utilizing and developing components: examples of this include PredictProtein (Yachdav et al., 2014), CATH (Sillitoe et al., 2013), Genome3D (Lewis et al., 2013), Reactome (Croft et al., 2014), Expression Atlas (Petryszak et al., 2014), Ensembl (Cunningham et al., 2015), InterMine (Smith et al., 2012), PolyMarker (Ramirez-Gonzalez et al., 2015) and the TGAC Browser (http://browser.tgac.ac.uk).

## Building and growing an open source community

In this section we discuss the factors that, we believe, have played an important role in the growth of the BioJS open source community.

### Clear mission and vision statements for the project

A vision is an ambitious aim that clearly states the value that the project adds. BioJS's vision is that ‘every online biological dataset in the world should be visualized with BioJS tools’. This vision statement communicates the way that BioJS aims to ‘change the world’. The members of our community are energized and motivated to contribute to this worthy cause. We also have a mission statement that describes what we do in order to achieve the our vision: ‘we develop an open-source library of JavaScript components to visualize biological data’. Whereas the vision is broad, ambitious and overarching, the mission statement is more practical and actionable. Having both a mission statement and a vision helps the BioJS community define what to do and who we are.

### Aligning interests with the wider community

BioJS aims to create the largest, most comprehensive repository of JavaScript-based tools that visualize online biological data. Many research groups that generate and make their data available will benefit if we are successful. Groups developing new components will now find that a host of core JavaScript functionality (I/O, parsers, basic visualization) is already available as part of the BioJS library. This creates an ecosystem where new contributors can plug into and extend existing components according to their needs and preferences. Furthermore, developers can leverage the visibility of the BioJS registry to increase the exposure of their own tools, and attract community adoption and feedback. Creating incentives for developers to add their tools into BioJS aligns the interest of many groups with our own project. This in turn has many benefits: (1) group leaders are more inclined to expend scarce resources that advance both their own and BioJS's goals; (2) the BioJS community grows as more participants find that their own goals are aligned with those of BioJS; and (3) some people who join the project may become long-term participants or even become evangelists who promote the project to others. Aligning interests with the community is crucial to grow contribution for projects that lack funding: indeed, the development of 27 components was done by groups that are not directly affiliated with BioJS, but who appreciated the value of contributing to BioJS and decided to integrate the components they had developed into the registry.

### Low technological barriers

Shared interests and goals may not be enough if contributing to a project involves significant and costly technical effort, so we have designed BioJS such that a potential contributor is faced with a relatively small set of technical requirements: s/he needs to know JavaScript, to ensure that their component conforms to the NPM package manager (https://www.npmjs.com), and to follow some simple naming conventions; contributors are not required to understand the core system. Moreover, multiple parallel contributions can be worked on at once, eliminating cross-development dependencies and affording contributors independence in creating their own components.

### Detailed and complete documentation and tutorials

The educational section of the BioJS website (http://edu.biojs.net) includes a comprehensive tutorial tailored for different types and levels of developers. Newcomers will find that the ‘getting started’ section sheds light on what the project is about and how core concepts (such as packaging) help to achieve modularity. Tutorials offer a step-by-step set of instructions on how to create a new BioJS component and detail the two-step process for publishing components. Contributors can then ‘graduate’ to the more advanced and detailed sections of the tutorial to explore the nuances of BioJS packages, components, the registry and the JavaScript technologies development stack. Good documentation is crucial for an open source project such as BioJS as it helps to ‘flatten’ the learning curve and thus promote participation, contribution and ultimately adoption.

### An open, fair and inclusive recognition strategy

BioJS's recognition strategy aims to be as inclusive and fair as possible. In 2013 we published an ‘Application Note’ in *Bioinformatics* including all contributing members, and in 2014 we published a collection of 14 articles in *F1000RESEARCH* that provided an update on the project at the time (Corpas, et al., 2014). By allowing publication of independent BioJS components as separate ‘Application Note’-type articles, we have enabled contributors who spend most of their time on voluntary basis to claim ownership and gain recognition for the work they do.

### Prompt and unfailingly positive communication

We have created several channels of communication to respond to needs of different project stakeholders. Our Gitter channel (https://gitter.im/biojs), which currently includes 47 members, is a public chat-room that is directly linked to our GitHub repository. Developers use the Gitter channel to announce new commits, bug fixes, patches and forks. Questions and answers about source code are posted to the room on a daily basis and, as a matter of principle, the core development team attempts to provide immediate responses on the Gitter channel. A timely response is crucial for keeping volunteers engaged and demonstrating the vitality of the project. The positive tone and friendly conduct on the chat room and mailing list also exemplify the inclusive ethos of the BioJS community.

### Early decentralization of responsibilities

Since its early days the BioJS community has adopted a culture that encourages and supports members who wish to assume direct responsibility for parts of the project. As a result, we do not have one central ‘benevolent dictator’, but rather a core group of motivated and committed community members.

### Promotion of the project through outreach and education

Time spent on promoting, evangelizing and networking is one of the most fruitful investments in the community. We regularly schedule tutorials, talks and meetings at events such as VizBi, ECCB, ISMB and its satellite meeting BOSC, as these are natural venues to connect with our target audience. BioJS first organized a half-day tutorial at the 2014 VizBi conference (held in Heidelberg, Germany) where 12 participants got hands-on experience in creating and adding new components to the registry. Later that year five coders travelled to Munich, Germany, to participate in the first ever BioJS hackathon, an event that gave birth to BioJS 2.0. Other hackathons, workshops and tutorials have been held in different venues across Europe and the US, aimed at increasing the visibility of the project and promoting contribution. During the winter semester of 2015 the Technical University of Munich ran an undergraduate level biological data visualization course in which 24 students were introduced to BioJS and contributed 11 components to the registry.

### Being ‘cool’ by joining community development programs

While many in science would prefer to just focus on traditional research activities, we discovered that joining high-profile programs, such as the Google Summer of Code (GSoC) program, was a highly effective way to advance the project goals. Such programs offer outstanding opportunities to interact with the best and the brightest students. The GSoC program offers student developers stipends to write code for various open source software projects, and BioJS received sponsorship for five GSoC internship positions in 2014. The impact of entering this program was immediate: we saw a sharp increase in the number of subscriptions to our community mailing list (from ∼40 members to nearly 100) in less than a month. The impact is also long lasting as we now have five peer-reviewed articles underway, describing projects that broaden the scope of the BioJS library and make it more useful for our community. Moreover, two of the students from the GSoC program (SW, DD) have now become members of the core group and, led by SW, have themselves orchestrated a series of hackathons in which BioJS was transitioned to version 2.0. The PhyloWidget project has also benefitted from the GSoC program (Jordan and Piel, 2008).

The BioJS project has also received support from the UK Software Sustainability Institute (http://www.software.ac.uk/open-call) to assess and help improve BioJS (by, e.g., evaluating documentation, code maintainability and the ease of development).

## Comparable approaches from other open-source communities

None of the points raised above are unique to BioJS as there are a number of other open source software projects in the life sciences. For instance, Cytoscape.js (http://js.cytoscape.org), an open source community developing a web-based viewer for the Cytoscape network viewer (Shannon, et al., 2003), is also mostly decentralized, being organized through some simple rules and with a community of contributors from both the public and private sectors. These contributors add new features to the library and discuss new feature ideas and bug reports. However, code contributions (i.e., pull requests) do require centralized review before merging into the main codebase.

The Galaxy project (Giardine, et al., 2005; Blankenberg, et al., 2010; Goecks, et al., 2010) is making advanced bioinformatics software accessible to biologists by directly providing an intuitive web interface to these applications. The Galaxy software enables the reproduction of computational research workflows by capturing and organizing the pipeline's layout and components into re-runnable protocols. The project also makes use of public collaboration tools to coordinate code contributions with the development community, and it maintains a dedicated portal (https://biostar.usegalaxy.org/) to communicate and support its user base. Like BioJS, the Galaxy core framework can be easily extended by plugins of all kinds, and the Galaxy core team offers the Galaxy ToolShed to distribute and share all these extensions in an App-Store like fashion. The annual Galaxy Community Conference (GCC) also attracts more than 200 participants and offers a variety of workshops and a hackathon.

The Bionode project (http://www.bionode.io) aims to provide modular, scalable and highly reusable tools for bioinformatics analysis. The community that surrounds this project is mainly composed of bioinformaticians and JavaScript (Node.js) developers who dedicate their time either as a hobby or because they need to add a feature (or fix a bug) for their own projects. Bionode is very open to any kind of contribution and does not enforce too many rules or governance to the community.

## Conclusions

Life science open source communities have their own special peculiarities and motivations. We have presented a compilation of experiences that, in our view, have helped BioJS to become a robust international project within a relatively short period of time. Apart from fostering the right skills and technical experience to develop BioJS, we have established formal means to reward contributors, to keep the community members motivated, and to increase our impact. If used with care and fairness, recognition can be a powerful lever with which to build communities. We believe that some of the lessons we have learned and the practices we have established will help others to build similarly robust open source projects and communities.
